# Assessing ChatGPT 4.0’s Capabilities in the United Kingdom Medical Licensing Examination (UKMLA): A Robust Categorical Analysis

**DOI:** 10.1038/s41598-025-97327-2

**Published:** 2025-04-15

**Authors:** Octavi Casals-Farre, Ravanth Baskaran, Aditya Singh, Harmeena Kaur, Tazim Ul Hoque, Andreia de Almeida, Marcus Coffey, Athanasios Hassoulas

**Affiliations:** 1 Centre for Medical Education (C4ME), School of Medicine, Cardiff University, Heath Park Campus, Cardiff, CF14 4YS United Kingdom; 2 University of Southampton School of Medicine, 12 University Rd, Southampton, SO17 1BJ United Kingdom; 3 Keele University School of Medicine, Keele University, University Road, Staffordshire, ST5 5BG United Kingdom; 4 OSCEazy Research Collaborative, Heath Park Campus, Cardiff, CF14 4YS United Kingdom; 5 HIVE Digital & Teaching Innovation Unit, University Hospital of Wales, 2nd Floor Office F-24 Heath Park, Cardiff, CF14 4XW United Kingdom; 6 Department of Urology, Southampton General Hospital NHS Foundation Trust, Tremona Road, Southampton, SO16 6YD United Kingdom

**Keywords:** ChatGPT, Medical student, Finals, Exam, Health services, Machine learning, Predictive medicine, Programming language

## Abstract

Advances in the various applications of artificial intelligence will have important implications for medical training and practice. The advances in ChatGPT-4 alongside the introduction of the medical licensing assessment (MLA) provide an opportunity to compare GPT-4’s medical competence against the expected level of a United Kingdom junior doctor and discuss its potential in clinical practice. Using 191 freely available questions in MLA style, we assessed GPT-4’s accuracy with and without offering multiple-choice options. We compared single and multi-step questions, which targeted different points in the clinical process, from diagnosis to management. A chi-squared test was used to assess statistical significance. GPT-4 scored 86.3% and 89.6% in papers one-and-two respectively. Without the multiple-choice options, GPT’s performance was 61.5% and 74.7% in papers one-and-two respectively. There was no significant difference between single and multistep questions, but GPT-4 answered ‘management’ questions significantly worse than ‘diagnosis’ questions with no multiple-choice options (*p* = 0.015). GPT-4’s accuracy across categories and question structures suggest that LLMs are competently able to process clinical scenarios but remain incapable of understanding these clinical scenarios. Large-Language-Models incorporated into practice alongside a trained practitioner may balance risk and benefit as the necessary robust testing on evolving tools is conducted.

**Supplementary Information:**

The online version contains supplementary material available at 10.1038/s41598-025-97327-2.

## Introduction

The release of the Open AI’s Large Language Model (LLM), ChatGPT (Generative Pre-trained Transformer) in November 2022 by Open AI sparked academic and commercial interest worldwide. ChatGPT Plus is the most powerful iteration to date and is supported by GPT-4, an LLM with billions of parameters altering its human language output^1^. Artificial Intelligence (AI) is poised to complement and evolve medical practice with possible applications covering every medical domain from data analysis to radiology reporting, medical education and even patient education and treatments such as evaluating embryo quality in In-Vitro Fertilisation (IVF) treatment^2–5^. A study of 1.5 million participants showed that LLMs are progressing rapidly and were nearly indistinguishable from humans when participants engaged with LLMs in a chat format^6^. However, LLMs are unable to display the key ‘human’ skills of empathy and intuition, limiting their function in patient communication^7,8^.

Currently, AI faces challenges in training data bias, reproducibility and reliability which limit its clinical implementation. Unpredictable errors, such as ‘hallucinations’, where ChatGPT may output coherent-sounding text with no factual support, can significantly mislead clinicians^9^. However, successful applications of AI within radiology and pathology suggest that a joint approach may improve efficiency and clinical outcomes^2,4^. Across medicine, AI may facilitate routine care elements such as patient summaries while integrating basic clinical knowledge into its approach^10^. Ultimately, AI may alleviate routine tasks from time-pressured medical staff and help them focus on more demanding tasks. At present, medical students and junior doctors in the United Kingdom (UK) lack the training and confidence to work with AI in their careers^3,11^. With certain medical schools already taking steps to implement Generative AI (GenAI) into literacy teachings, medical school curricula must adapt to safely promote AI as a powerful clinical tool^12^.

The previous iteration of ChatGPT, an OpenAI ChatBot powered by GPT-3.5 was able to pass the US Medical Licensing Exams (USMLE), the benchmark for medical practice in the United States^13^. ChatGPT’s ability to accurately interpret complex clinical scenarios reinforced ideas of incorporating similar AI models into medical practice. Improvements to GPT outlined in the pilot paper for GPT-4 call for updates to its performance in medical scenarios. OpenAI states that on a medical self-assessment program, GPT-4 scores 75% relative to GPT-3.5’s score of 53% ^1^. The 41% improvement may represent an increase in the utility of AI tools for support for junior doctors, but ethical and practical concerns limit GPT’s potential in the clinical setting. Thus, to prevent misuse of ChatGPT, it is important to determine whether it can perform at the minimum level of a newly qualified doctor.

In 2024, the General Medical Council (GMC) will introduce the medical licensing assessment (MLA) nationwide to set the clinical knowledge benchmark for foundation-year doctors^14^. The exam, consisting of 200 multiple-choice questions across two papers will centre around clinical vignettes targeting the entire patient journey. We hypothesise that ChatGPT can perform at a passing standard for a new junior doctor. Research conducted by Lai et al.., broadly assessed the performance of ChatGPT in the mock UKMLA papers, exploring its role in medical education^15^. This study aims to further assess ChatGPT’s accuracy in the UKMLA and stratify performance by question type to identify the range of tasks within which ChatGPT may reliably supplement current clinical practice. We aim to conduct an in-depth analysis of ChatGPT’s (i) accuracy in answering the UKMLA paper with and without providing multiple-choice answers; (ii) ability to answer one-step versus multi-step questions; and (iii) theme-based answering abilities.

## Methods

### Medical question bank sets

We sampled questions from the Medical Schools Council (MSC) site, which offered the only available MLA mock papers (accessed 10/06/2023). The exam tests 24 areas of clinical practice split across two 100-question papers, using clinical scenarios as question stems, and providing five answer options^14^. These exam papers are freely available and so allow a fixed point of comparison for future releases of GPT or alternative AI tools. From the 200 questions, nine contained images which GPT could not interpret leaving a total of 191 questions.

### Prompting ChatGPT

We prompted ChatGPT with 191 multiple-choice questions (MCQs). Prompts were formatted by placing the scenario first, followed by the question on a new line. We tested the performance of ChatGPT with and without the MCQ options within the prompts. When including the MCQ options in the prompt, they were separated from the question by another blank line.

### Data collection

All data was collected between June 10th and June 15th, 2023. Questions were correct if they matched the answers given by the MSC. All answers were placed onto a shared spreadsheet to allow data to be reviewed.

We first divided the questions by whether they were either single-step or multistep questions. Multi-step questions required two or more stages of working, such as deducing a diagnosis from the vignette before deciding on the management. Determining the number of steps in questions was carried out separately by two assessors, and an independent third party reviewed disputed questions.

We later analysed the strength of ChatGPT across all questions and within the subcategories of topic and question type. The questions were split by broad speciality (Table 1). A third independent party reviewed any disputes over question categorisation.

**Table 1:**
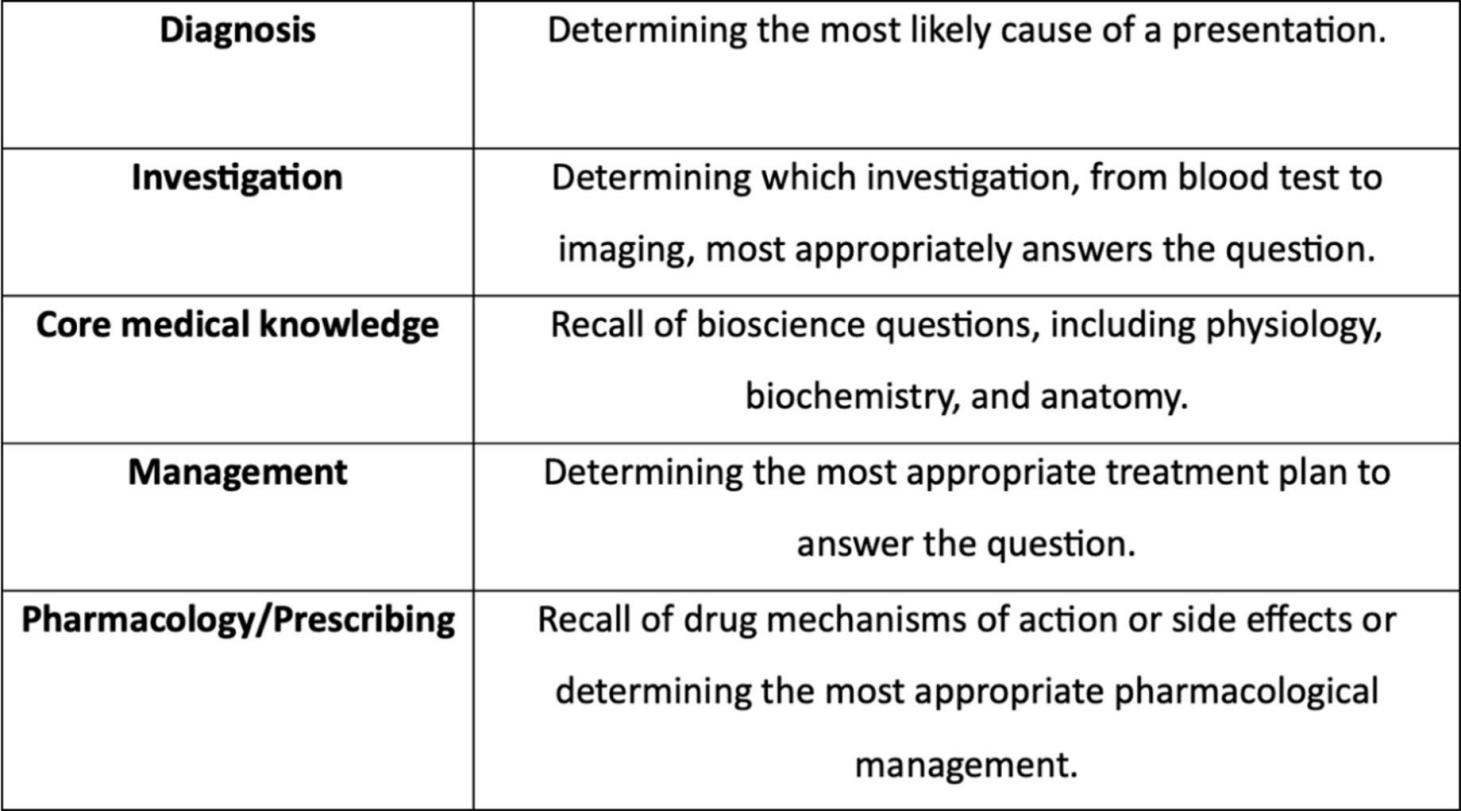
Categories of question types are used for subdividing questions.

Answers produced by Chat-GPT were categorised into three domains:

1. Accurate: single correct answer.
2. Indeterminate: a correct answer embedded within multiple alternative answers.
3. Incorrect: no specific answer or incorrect answer.

Question topics and notes on each question were input alongside answer accuracy on a shared spreadsheet.

### Data analysis

We analysed the data using SPSS (version 29). A chi-squared test was used to assess the association between the question style used as the input and ChatGPT’s categorised output. Results were further analysed using an Independent T-test to evaluate the association between results with and without MCQ prompts.

## Results

### Overall performance

We primarily hypothesised that ChatGPT’s would perform better when given multiple-choice options with secondary hypotheses including a better performance in single-step questions and questions that assess diagnostic competence.

**Fig. 1:**
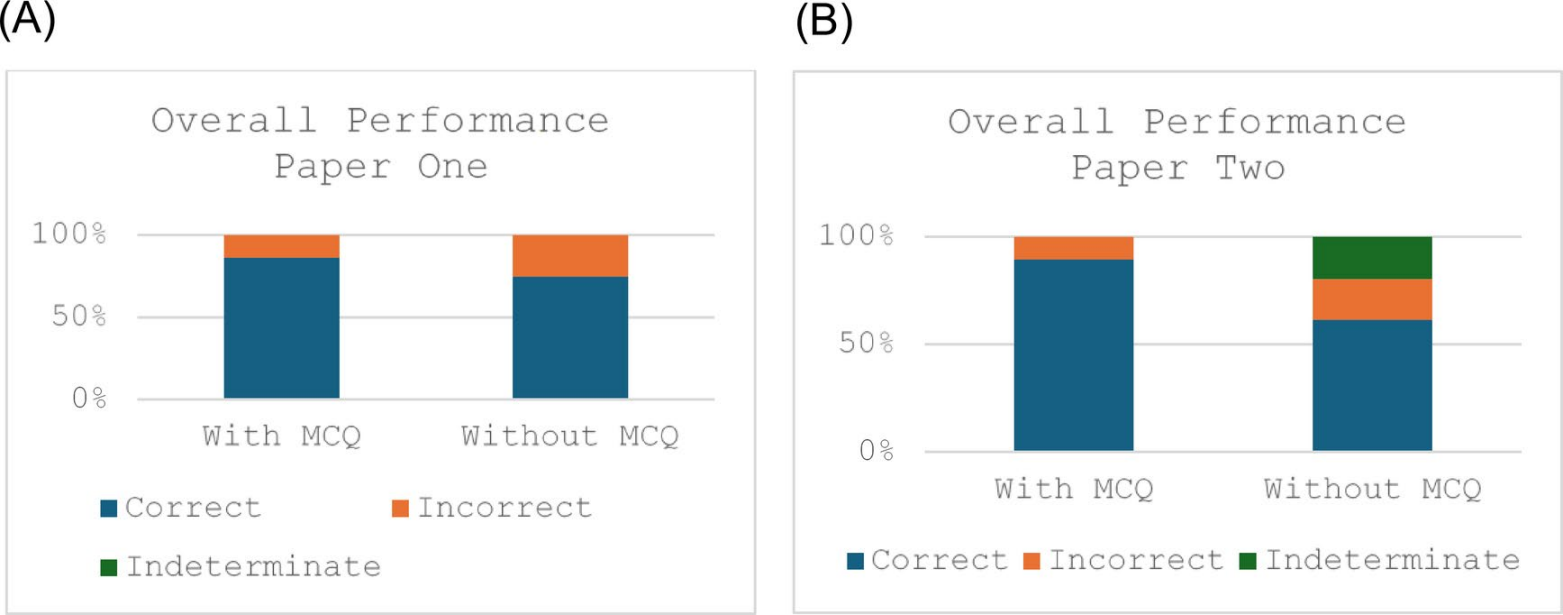
Bar chart showing the accuracy of GPT both with and without the MCQ prompt for (A) paper one and (B) paper two.

ChatGPT scored 86.3% (82/95) in paper one and 89.6% (86/96) in paper two. ChatGPT’s performance decreased to 61.5% without the multiple-choice options in paper one and 74.7% in paper two (Fig. 1 (A) and (B)). There were no indeterminate answers when options were provided. A Chi-squared test comparing results with and without MCQ prompt across both papers produced a p-value of 0.007 (N-191, X^2^ = 10.02), suggesting high statistical significance.

GPT answered eight questions accurately without prompts but inaccurately when presented with the MCQ options. All other answers were correct when provided with the MCQ prompt following an incorrect answer with no prompt.

### Question type

**Fig. 2:**
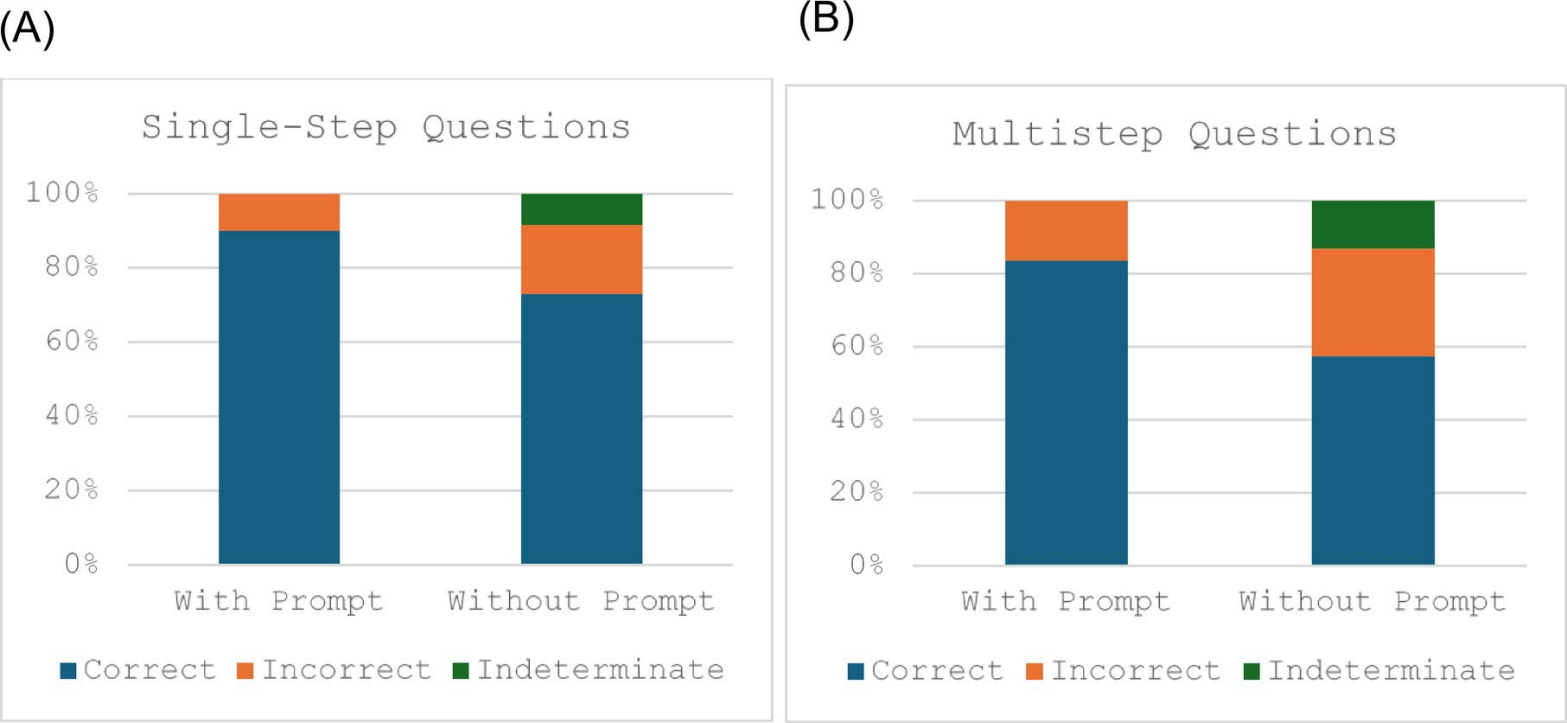
Bar chart showing answer outcomes with and without the MCQ prompt for (A) single-step and (B) multistep questions.

The exams consisted of 130 single-step and 61 multistep questions. ChatGPT correctly answered 90% (117/130) single-step questions and 83.6% (51/61) multistep questions when presented with an MCQ prompt. ChatGPT correctly answered 73.1% (95/130) single-step questions and 57.4% (35/61) multistep questions when presented with no MCQ options (Fig. 2(A) and (B)).

A chi-square test revealed a statistically significant association between the accuracy of results for one-step vs. multi-step question outcomes without the MCQ prompt, *X*^2^ (*N* = 191) = 11.14, *p* = 0.025. The results for single vs. multistep questions with the MCQ prompt yielded a non-significant association between the categories *X*^2^ (*N* = 191) = 2.63, *p* = 0.268.

### Competency

ChatGPT answered ‘diagnosis’ questions with MCQ options with the highest accuracy, scoring 91.2%. There were no indeterminate answers when MCQ prompts were provided. The most indeterminate responses were ‘pharmacology’ questions, with GPT missing 18.8% of the answers.

**Fig. 3:**
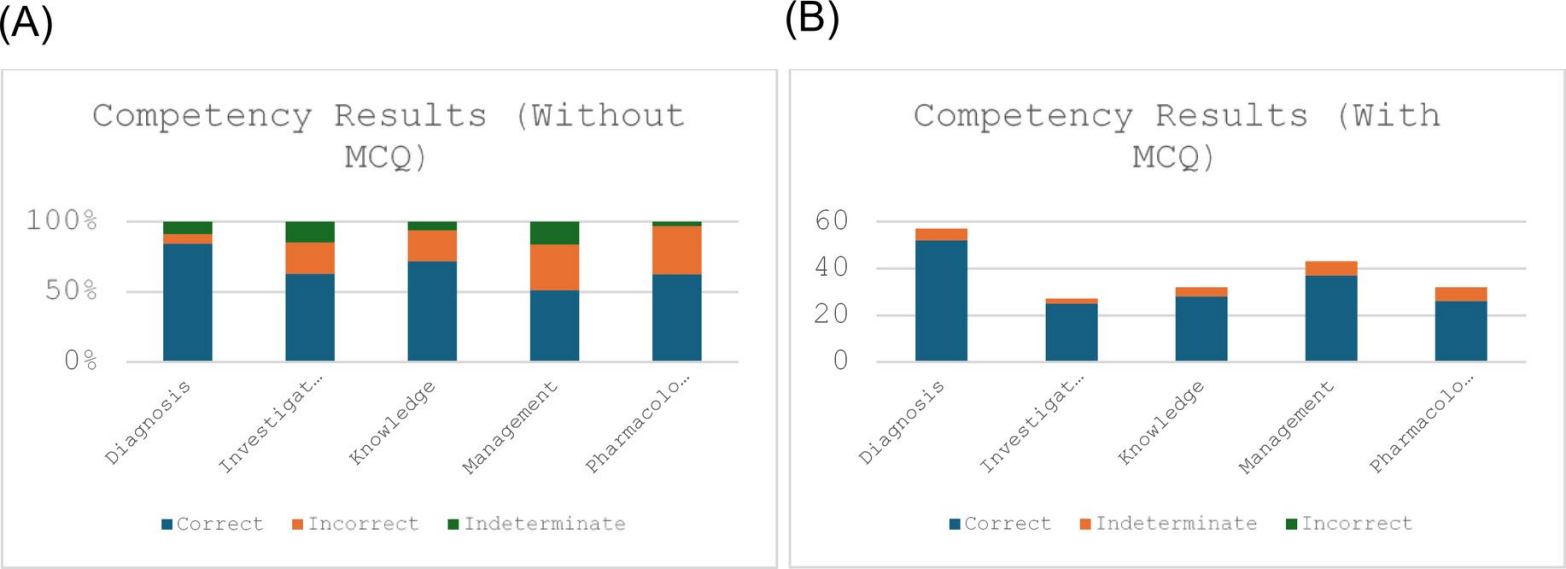
Bar charts showing the distribution of correct, incorrect, and indeterminate answers across all different competencies (A) without MCQ options and (B) with MCQ options.

Without an MCQ prompt, the highest accuracy was again in ‘diagnosis’ questions with 84.2% accurate answers. The highest error rate was in responses to ‘management’ questions with only 51.2% correct, 32.6% indeterminate, and 16.2% incorrect answers. The most indeterminate answers were again in the ‘pharmacology’ subsection with 34.4% indeterminate answers, and the least indeterminates at 7.0% were answered in ‘diagnosis’ questions (Table 2 (A), (B) and Fig. 3 (A), (B)).

**Table 2:**
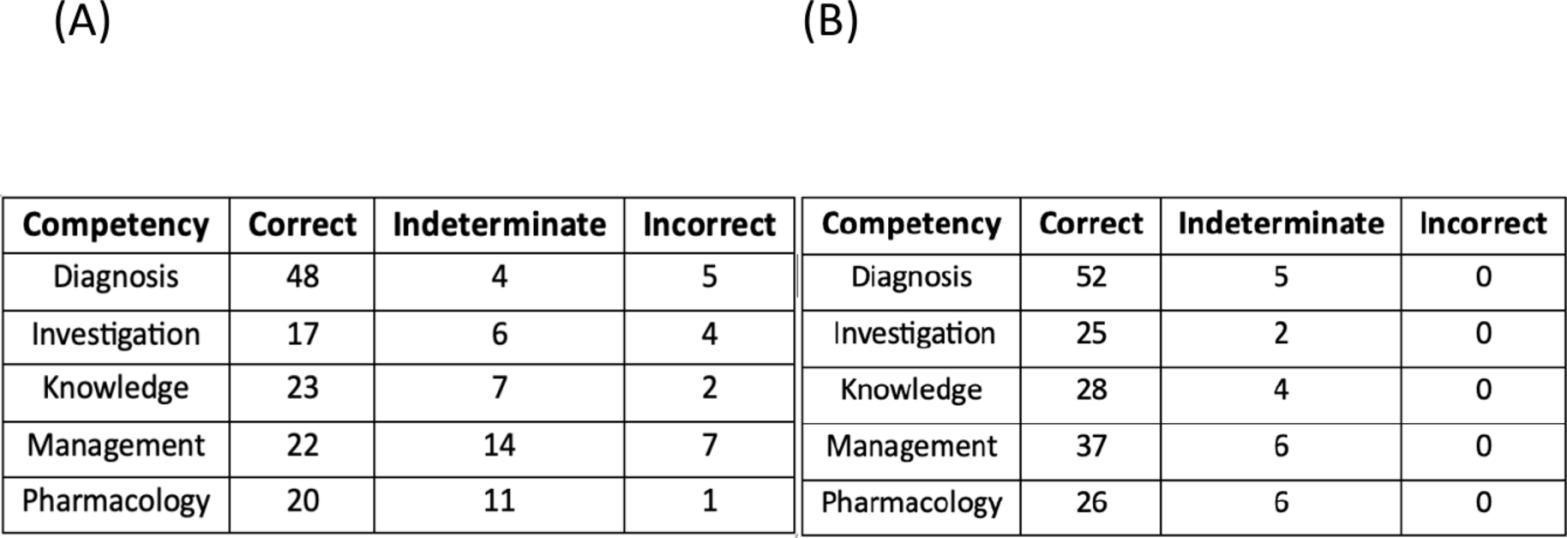
(A) shows the distribution of answers for each competency without the MCQ options provided. (B) shows the distribution of answers for each competency with the MCQ options provided.

A chi-square test of independence showed that there was a significant association between different competencies when tested without the MCQ prompt: *χ2* (*N* = 191) = 18.93, *p* = 0.015, however, there was no significant association when tested with the MCQ prompt: X2 (2, *N* = 191) = 2.64, *p* = 0.620.

## Discussion

To our knowledge, we have conducted the first study looking at GPT-4’s performance in the MLA testing format focusing on specific competencies, question types and the provision of an MCQ prompt. Overall, GPT-4 performed at a passing standard for a final-year student undertaking the MLA. The presence of multiple-choice options removed uncertainty and strengthened the accuracy of the tool, whereas there was a global decline in performance when answering open-ended questions. GPT-4 only demonstrated significant differences in accuracy when answering open-ended questions targeted at different points within the patient journey.

Kung et al.’s study showed that ChatGPT could pass the USMLE, and since then, both the strength of AI and the format of UK medical school final exams have significantly changed^13^. Studies by Lai et al. and Al-Shakarchi et al. have been conducted on ChatGPT’s efficacy in passing the new UKMLA. Lai et al.’s study demonstrated that ChatGPT is capable of passing the UKMLA but concludes it is most suited as a supplementary, monitoring or learning tool rather than inpatient diagnosis or interaction^15,16^. Al-Shakarchi et al.’s study analyses questions using a specialty-based approach suggesting that ChatGPT can perform well applying simple deductive reasoning to its vast data set^16^. However, ChatGPT was found to overlook fine details in medical knowledge-based decisions and lacked patient interaction skills and the ability to diagnose and holistically manage cases with accurate prescribing skills^17^. Overall, ChatGPT appears limited as an independent tool and requires significant supervision to ensure optimal patient-specific decisions and interactions.

With more powerful AI, it follows that AI should answer medical scenarios with improved accuracy and thus pass the MLA, the UK’s parallel to the USMLE. Indeed, GPT-4’s score of 86.3% and 89.6% in papers one and two respectively places the AI beyond the average performance of a medical student. Furthermore, we found no significant differences in the performance of GPT-4 across different topics. Strength in breadth of knowledge supports an LLM’s incorporation into clinical practice as an aid to increase junior doctors’ diagnostic sensitivity.

Maitland et al. tested LLM’s ability to pass the Membership of the Royal College of Physicians (MRCP) part 1 and 2 examination questions, increasing the required depth of knowledge from the LLM and nevertheless showed that it far exceeded the required pass mark^18^. However, analysis of ChatGPT’s mistakes suggests that the LLM focused on general cues in the vignette while potentially neglecting important nuances. LLM’s breadth over depth of knowledge cautions against use in applications and technologies with patient-specific medical impact. Importantly, Maitland et al. recommend that errors should be fully understood before ChatGPT is utilized to supplement clinical practice^18^.

In the clinical environment, there are no set options for the AI to choose from. When multiple-choice options were removed and GPT-4 was prompted to independently determine the best answer, GPT − 4 showed a clear reduction in accuracy across papers one (-24.8%) and two (-14.9%). This error rate was amplified when the complexity of open-ended questions was increased, as ChatGPT had a 15.7% lesser average accuracy while answering multistep questions, as compared to single step. Despite question stems asking for a single best answer, we found that GPT-4 began to provide a greater proportion of ‘indeterminate’ responses, especially when tackling patient pharmacology scenarios. However, inconclusive answers may prove detrimental in an exam format, but many patient and service provider factors alter the approach to managing patients, and a definitive answer may prematurely focus the treatment and prove detrimental to the patient’s care. Acknowledging uncertainty or variation in the evidence and presenting multiple options may serve junior doctors better by giving general guidance before allowing clinician-led final decisions.

Distractors in clinical scenarios may highlight further gaps in GPT-4’s suitability for clinical practice. Counterintuitively, eight open-ended questions were answered correctly by the AI and were ‘indeterminate’ with multiple-choice options provided. Plausible alternatives and red herrings may impact GPT-4’s evaluation of answers and practitioners must be cautious of the reasonable and thus convincing errors in diagnosis and treatment AI can suggest. Nuances in vocabulary and patterns in the exam questions’ phrasing are familiar to junior doctors, but AI may require more training before reaching equivalence. By extension, the many distractors in practice will require thorough filtering, a process inherent to taking a good clinical history. Furthermore, GPT-4’s output may produce convincing but entirely false suggestions in the well-described ‘hallucination’ phenomenon^9,19,20^. LLMs may create a linguistically coherent paragraph on any question prompt by combining information from multitudes of sources and thus derive a sequence of words by using probabilities rather than analysis of the question prompt^21^. In questions with multiple options, GPT was able to match question stems to training data and select the response closest to information in its data set but when the same questions were asked in open-ended form, ChatGPT could not match terminology or produce significantly right answers resulting in wrong or inconclusive answers.

Importantly, GPT’s equivalence, or even superiority, to junior doctors when answering MCQ questions does not supersede all competencies required for a student to graduate into clinical practice. Objective or Integrated Structured Clinical Examinations (OSCEs or ISCEs, respectively) evaluate a student’s communication, patient examination, and data interpretation abilities, culminating in a test of clinical reasoning by deriving diagnoses and management plans. While ChatGPT and other LLMs might excel in input-based cues by harnessing training datasets, their inability to detect and integrate social, verbal, and visual cues restricts LLMs from being supplementary tools rather than operating with minimal physician input.

GPT remains limited by the standard of its training data. Despite impressive results, analysis of GPT’s performance across stages of the clinical process showed a significant decrease in performance in the ‘management’ questions from the ‘diagnosis’ scenarios. This suggests to us that while GPT can understand from its training data the clinical signs and symptoms that lead to a diagnosis, it is not completely able to correlate the demands of the questions and hence subsequently not be able to address the management aspects that certain questions ask for. GPT remains restricted to its training data. Despite this training data being broad, this study shows that GPT requires more training data input and needs to learn from user information to better equip its data and better demonstrate this in such prompts as seen in this study. Simultaneously, the management of conditions improves as new research challenges current practice, but patient presentations remain comparatively constant across many pathologies. Thus, GPT’s relative ease in making diagnoses reflects that of medical practitioners and keeping up to date to ensure optimal patient treatment requires LLMs to be trained on relevant data. As guidelines continue to vary by hospital trust within the National Health Service (NHS), LLMs will require continuous and rigorous regional training to ensure consistency in patient care before application in practice. Storage and computational power may prove restrictive to applying AI in a rapidly evolving field such as medicine.

Ethical dilemmas may also hinder the application of AI in clinical medicine. Medical research often underrepresents minority populations, and thus its results cannot be generalised to train AI-based models^10^. Diagnostic errors made by LLMs due to insufficient or flawed training could detrimentally influence clinical decisions. Furthermore, LLM black box algorithms obscure its decision-making process so clinicians cannot appraise the model’s process and output^22^. Additionally, care must be taken to prevent the depersonalisation of care that patients may experience and the deskilling of healthcare professionals due to an overreliance on AI. No margin of error can be made when making patient care decisions; practitioners must apply caution when incorporating LLM outputs within medical practice.

Flaws in GPT-4’s performance likely result from outdated, globally acquired, unverified training data, not specific to medicine^1^. Simultaneously, the diversity of the training data makes GPT’s ability to answer a range of topics and question structures in the MLA remarkable. Pairing GPT-4’s knowledge with improvements enabling GPT-4 to interpret clinical images, such as cardiac monitoring and X-rays, will further broaden its clinical application and potential as a screening tool. Additionally, in an era of big data, unique variables such as patient’s genetic profiles will soon influence clinical decisions. AI’s ability to integrate expanding quantities of data may identify optimal treatment strategies for patients, help structure complex summaries or flag abnormalities buried in years of patient documents and results. Herein lies an exciting potential to harness the power of LLMs within medicine, but analysis of LLMs’ ability to extract all salient details from patient histories is required to ensure safe and reliable outputs.

A rapidly evolving application of ChatGPT can be found in the medical education field. Khan et al. highlight ChatGPT’s utility in assisted teaching, automating scoring systems, personalised learning, research summarization, and generating clinical scenarios^23^. Many medical education platforms, question banks and OSCE practice tools are focussing on the integration of AI into producing questions and simulated patients, a typically labour-intensive process. As the role of AI becomes clearly defined within medical education, new generations of clinicians will use AI-enhanced tools to train for practice^24^.

### Limitations

Limitations exist within this study’s design. We studied GPT-4s applicability to clinical practice using an exam with carefully designed and topic-specific questions. Naturally, exam questions cannot reflect reality, where relevant clinical information must be paired with a breadth of investigations to decide the diagnosis or management plan. Furthermore, GPT-4 is not trained on the UK-specific guidelines which dictate the MLA’s correct answers. Thus, a model trained using information consistent with the assessment may improve its score, and a sample size greater than 200 will better highlight the key deficiencies of LLMs applied to medical scenarios. Furthermore, despite appraisals of questions being single-versus-multi step and responses by ChatGPT being ranked as correct, incorrect and indeterminate the data collection was conducted by multiple data collectors and hence, there could be interpretation bias introduced by these multi-layered review process that skewed data reporting. However, this was minimized by any conflict being resolved by the senior authors.

The sample size of questions used remained small at 200 questions (with 191 questions included). This served as a limitation as publicly available UKMLA questions were limited with major question banks contacted declining for usage of their questions. Furthermore, at the time of data collection, due to LLMs not having the ability to analyze scan or picture-based data, certain questions had to be excluded. The exclusion of such questions might not provide a holistic picture of the UKMLA assessment and by extension might decrease the generalizability of our conclusions.

Despite LLMs such as ChatGPT having access and being trained on publicly available information on the internet, licensed information from third parties and information provided by users, there still exists a limitation to the LLM’s dataset. Without being able to see, depict and understand visual and video clinical signs, ChatGPT’s training data remains limited with regards to clinical knowledge. This might affect or interfere with its ability to answer questions accurately with a holistic clinical picture in mind.

Additionally, inputting open-ended questions first followed by MCQ-included questions could introduce ChatGPT’s answering bias. ChatGPT is an LLM that is meant to learn from in-context dialogued human feedback and hence, we cannot ascertain the extent to which the initial question input influenced the answers it selected when inputting questions with MCQs^25^.

## Conclusion

We show that GPT-4 can use a sophisticated set of algorithms to provide a prompt-based response required to pass a medical school final examination. However, regular updates and widespread variation in practice likely underpinned the errors in LLM answers. Integration of novel technologies is inevitable, but ensuring ethical and responsible use is critical for patient care. Using evidence-based and up-to-date information to train new LLMs may reduce the myriad of digital resources into software capable of digesting information quickly and accurately and could enhance efficiency in clinical practice. The experience of a trained physician cannot currently be replicated, but LLMs may add another tool to the expanding toolkit of the modern clinician.

## Electronic supplementary material

Below is the link to the electronic supplementary material.

Supplementary Material 1

## Data Availability

Data has been provided within the supplementary information files.
